# Podocyte Vps34 deficiency drives early-onset glomerulopathy and mesangial IgA-dominant immune-complex deposition: a novel mouse model linking vesicular trafficking to glomerular immune dysregulation

**DOI:** 10.3389/fimmu.2026.1936691

**Published:** 2026-09-09

**Authors:** Shu Qu, Ting Gan, Ting-hui Qu, Shang Zeng, Yang Li, Ji-cheng Lv, Hong Zhang, Hou-hua Li, Hui Wang, Xu-jie Zhou

**Affiliations:** 1 Renal Division, Department of Medicine, Peking University First Hospital, Beijing, China; Peking University Institute of Nephrology, Peking University, Beijing, China; Key Laboratory of Renal Disease, Ministry of Health of China, Beijing, China; Key Laboratory of Chronic Kidney Disease Prevention and Treatment (Peking University), Beijing, China; 2 State Key Laboratory of Natural and Biomimetic Drugs, Peking University, and School of Pharmaceutical Science, Peking University, Beijing, China; 3 Department of Pathology, School of Basic Medical Sciences, Peking University Third Hospital, Peking University Health Science Center, Beijing, China

**Keywords:** IgA nephropathy, immune-complex glomerulopathy, mesangial IgA deposition, podocyte, proteinuria, VPS34

## Abstract

**Background:**

Podocytes are indispensable for maintaining glomerular filtration barrier integrity and immune homeostasis. Although vacuolar protein sorting 34 (Vps34), a class III phosphoinositide 3-kinase, orchestrates intracellular membrane trafficking, its roles in glomerular immune regulation remain incompletely defined. This study identifies a critical link between podocyte-specific Vps34 dysfunction and glomerular immune injury, characterized by pronounced mesangial IgA deposition.

**Methods:**

A podocyte-selective Vps34 knockout mouse model was generated. Kidney architecture and function were systematically assessed using histological, immunofluorescent, and ultrastructural analyses. Systemic immunoglobulin concentrations were measured, and bulk renal transcriptomics were conducted to delineate molecular alterations.

**Results:**

Selective deletion of Vps34 in podocytes induced rapid-onset proteinuria from three weeks of age, progressive renal failure, and early mortality (median survival nine weeks). These mice exhibited marked mesangial expansion, focal and global glomerulosclerosis, and widespread foot process effacement. Immunofluorescence revealed intense mesangial IgA deposition, accompanied by elevated serum IgA and IgM and reduced IgG levels. Comprehensive transcriptomic profiling revealed progressive immune-related transcriptional changes, highlighting upregulation of antigen presentation, B cell activation, cytokine signaling, and extracellular matrix remodeling. In addition, Vps34 deficiency was associated with increased expression of DAMP-associated and inflammasome-related genes, suggesting engagement of inflammation-related transcriptional programs within the injured kidney.

**Conclusions:**

Podocyte-specific Vps34 deficiency leads to progressive podocyte injury accompanied by IgA-dominant immune-complex deposition and immune-related changes in the kidney. These findings reveal an association between podocyte dysfunction and glomerular immune abnormalities and suggest a potential role of vesicular trafficking defects in immune dysregulation during proteinuric kidney disease.

## 1 Introduction

The kidney’s glomerular filtration barrier is vital for maintaining metabolic balance and immune homeostasis. Podocytes, as highly specialized epithelial cells lining the glomerular basement membrane, play a central role in supporting filtration integrity by orchestrating dynamic cytoskeletal and vesicular processes (1). Their adaptive membrane turnover and endocytic recycling are essential to respond to fluctuating physiological stresses and prevent glomerular injury (2–5).

Vacuolar protein sorting 34 (*Vps34*; also known as PIK3C3) is a class III phosphoinositide 3-kinase fundamental to autophagy initiation and vesicular trafficking, and its global loss is embryonically lethal. Conditional ablation of *Vps34* in organs such as the brain, liver, and heart has revealed distinct tissue-specific physiological roles, particularly in regulating membrane dynamics, autophagosome formation, and intracellular signaling (6–10). In podocytes, Vps34 deficiency has been shown to disrupt autophagic flux and endolysosomal homeostasis, resulting in rapid-onset proteinuria, podocyte vacuolization, and progressive glomerulosclerosis (6, 11). However, previous studies have primarily focused on the cell-intrinsic consequences of Vps34 loss in podocytes, and whether impaired podocyte vesicular trafficking can influence the glomerular immune microenvironment and immune-complex deposition remains largely unknown.

Emerging evidence highlights complex crosstalk between podocyte injury and immune mechanisms in kidney disease, with podocyte dysfunction recognized not only as a consequence of immune-mediated glomerulopathies, but potentially a trigger for aberrant immune activation and mesangial remodeling (12, 13). However, the precise mechanisms linking podocyte-intrinsic defects to glomerular immune abnormalities are not fully understood, representing a critical gap in renal immunology and pathobiology.

In this context, our study investigates whether podocyte-specific Vps34 deficiency is associated not only with the cell-autonomous podocyte injury previously described in Vps34-deficient podocytes (7, 10, 11), but also with alterations in the glomerular immune environment. By combining time-course histopathological assessment, ultrastructural analysis, immunofluorescence, systemic immunoglobulin profiling, and bulk RNA sequencing of whole kidneys in a podocyte-selective Pik3c3 knockout mouse, we identify a previously unrecognized phenotype characterized by podocyte injury accompanied by mesangial IgA-dominant immune-complex deposition, complement co-deposition, and progressive changes in immune- and extracellular matrix-related gene expression programs. These findings extend the recognized role of podocyte Vps34 beyond cell-intrinsic homeostasis and suggest a potential link between podocyte dysfunction and glomerular immune abnormalities. This model may provide an opportunity to further investigate the relationship between podocyte injury and mesangial immune-complex accumulation in proteinuric kidney disease.

## 2 Materials and methods

### 2.1 Generation of podocyte-specific *Vps34* knockout mice

Podocyte-specific *Vps34* knockout (mVps34^pdKO^) mice were generated by crossing Pik3c3^flox/flox^ mice with Nphs2-Cre transgenic mice (Jackson Laboratory, B6.Cg-Tg (NPHS2-Cre)295Lbh/J), enabling targeted deletion of exon 4 in the *Pik3c3* gene within podocytes. The floxed *Pik3c3* allele was engineered using CRISPR/Cas9-mediated loxP site insertion (Cyagen Biosciences, China). Littermates lacking Cre recombinase (Pik3c3^flox/flox^) served as controls. All animals were maintained under specific pathogen-free (SPF) conditions on a C57BL/6J background. Genotyping was performed on tail-derived genomic DNA, and primer sequences are listed in **Supplementary Table 1**.

Both male and female mice were included at each time point in approximately equal proportions. Sex was recorded and reported descriptively, as the study was not designed to detect sex-specific effects. Mice were allocated to predefined time-point cohorts based on litter availability and cage organization, without *post-hoc* selection based on phenotype. Littermate Pik3c3^flox/flox; Nphs2-Cre− mice served as the primary control group, and Pik3c3^flox/+; Nphs2-Cre+ mice were included as an additional genotype group at each time point.

### 2.2 Survival and renal function assessment

Experimental cohorts (n = 54) were monitored longitudinally for body weight and survival from weaning until study endpoint. Blood urea nitrogen (BUN) and urinary creatinine concentrations were determined using commercial ELISA kits (Bioassay Systems, DIUR-100; DICT-500) following manufacturer protocols. Urinary albumin excretion was measured by a mouse-specific ELISA (Immunology Consultants Laboratory, E-90AL). Urine samples from all genotypes were obtained from metabolic cages under controlled conditions, with hematuria screened via standardized dipstick analysis (URIT Medical Electronic Co., Ltd.). Glomerular filtration rate (GFR) was assessed by transcutaneous FITC-sinistrin clearance using the MediBeacon system (MediBeacon, Mannheim, Germany) in conscious mice as previously described (30). The elimination half-life of FITC-sinistrin (t_1_/_2_) was determined from the fluorescence decay curve using MediBeacon Studio software, and GFR values were calculated according to the software algorithm and expressed as ml·min^–1^·100 g^–1^ body weight after normalization to body weight.

### 2.3 Tissue processing, histopathology, and immunofluorescence

Kidneys were excised and processed either by fixation in 4% paraformaldehyde followed by paraffin embedding, or by immediate snap-freezing in OCT compound for cryosectioning. Paraffin-embedded tissue sections (3 μm) underwent staining with hematoxylin and eosin (H&E), periodic acid–Schiff (PAS), and Masson’s trichrome, adhering to standard protocols. For immunofluorescence studies, frozen cryosections were incubated with primary antibodies specific to mouse IgA, IgG, IgM, and complement C3. Nuclear counterstaining was performed using DAPI to delineate cellular architecture. All staining procedures incorporated established positive and negative controls to ensure specificity and reproducibility. Histological grading (mesangial matrix expansion, percentage of sclerotic glomeruli, and interstitial fibrosis) and semi-quantitative immunofluorescence scoring (IgA, IgM, and C3 deposition) were performed by an observer blinded to genotype. Mesangial matrix expansion was quantified on PAS-stained sections using ImageJ-based area segmentation and expressed as the percentage of mesangial matrix area per glomerulus (≥15 glomeruli per mouse). The percentage of sclerotic glomeruli (segmental and global sclerosis) was determined on the same sections. Glomerular IgA, IgM, and C3 deposition was semi-quantitatively scored on a 0–4 scale (0 = absent, 1 = trace/segmental faint, 2 = mild diffuse, 3 = moderate diffuse, 4 = strong diffuse) by a blinded observer. For each animal, 15 consecutive cortical glomeruli without global sclerosis were scored, and the per-animal mean score was used as the statistical unit. Between-group comparisons were performed using the two-tailed Mann–Whitney U test.

### 2.4 Serum immunoglobulin quantification

Serum concentrations of IgA, IgG, and IgM, together with albumin and total protein, were quantified using isotype- and analyte-specific sandwich enzyme-linked immunosorbent assays (ELISAs) with commercially available kits according to the manufacturers’ instructions. Calibration curves and appropriate controls were included in each assay. Serum IgA, IgG, IgM, and albumin concentrations were reported as absolute values. Immunoglobulin-to-albumin ratios (IgA/Alb, IgG/Alb, and IgM/Alb) and inter-isotype ratios (IgA/IgM and IgG/IgM) were additionally calculated.

### 2.5 Transmission electron microscopy

Renal cortical tissue specimens were immediately immersion-fixed in 2.5% glutaraldehyde prepared in 0.1 M phosphate buffer (pH 7.4) and incubated overnight at 4 °C to ensure optimal ultrastructural preservation. Following primary fixation, samples were post-fixed in 1% osmium tetroxide, sequentially dehydrated through graded ethanol series, and infiltrated with epoxy resin. Ultrathin sections (70 nm thickness) were cut using an ultramicrotome and subsequently stained with uranyl acetate and lead citrate. Electron micrographs were acquired using a JEM-1400 transmission electron microscope (JEOL, Tokyo, Japan) according to manufacturer protocols.

### 2.6 RNA isolation and bulk RNA sequencing

Total RNA was extracted from whole kidney tissue using TRIzol reagent (Thermo Fisher Scientific), strictly adhering to the manufacturer’s protocols to ensure RNA integrity. RNA quality and concentration were assessed using the RNA Nano 6000 Assay Kit and Agilent 2100 Bioanalyzer (Agilent Technologies). Strand-specific mRNA sequencing libraries were constructed with the Hieff NGS Ultima Dual-mode mRNA Library Prep Kit (Yeasen Biotechnology) according to the recommended workflow. Sequencing was carried out on the Illumina NovaSeq 6000 platform, generating 150 bp paired-end reads. High-quality sequencing reads were aligned to the mouse reference genome using HISAT2 (14). Quantitative differential gene expression analysis was performed with the DESeq2 package in R, defining significant differentially expressed genes (DEGs) as those with a fold change ≥1.5 and a false discovery rate (FDR) <0.05. Functional enrichment analyses for DEGs were conducted using the KOBAS database (15, 16) and visualized using clusterProfiler (17) in R.

### 2.7 Statistical analysis

All statistical analyses were performed using IBM SPSS Statistics version 26. Continuous variables are reported as mean ± standard error of the mean (SEM). Comparisons between two groups were conducted using unpaired, two-tailed Student’s t-tests, while analyses involving more than two groups were evaluated using one-way analysis of variance (ANOVA) followed by Tukey’s *post hoc* test for multiple comparisons. Survival outcomes were analyzed by Kaplan-Meier methodology with statistical significance determined via the log-rank test. A p-value less than 0.05 was considered indicative of statistical significance.

The individual mouse was considered the biological unit for all statistical analyses. For histopathological and immunofluorescence analyses, multiple glomeruli or fields from each mouse were quantified, averaged, and the per-animal mean value was used for between-group comparisons. Except for survival analysis and body-weight measurements collected longitudinally within the same cohort, other measurements at each time point were obtained from independent terminal cross-sectional cohorts. Therefore, comparisons among genotypes at each time point were performed using unpaired two-tailed Student’s t-tests (with Welch’s correction when appropriate) or one-way ANOVA with Tukey’s *post hoc* test. Semi-quantitative immunofluorescence scores were analyzed using the two-tailed Mann–Whitney U test.

For longitudinal body-weight measurements, data were additionally analyzed using a linear mixed-effects model with mouse as a random effect and genotype and age as fixed effects.

No *a priori* power calculation was performed because this was a newly established mouse model. Sample sizes (typically n = 6–13 per genotype per time point) were determined based on previous Vps34 podocyte-specific knockout studies and cohort availability. Pre-specified exclusion criteria were limited to technical failures, including failed genotyping, sample collection failure, or assay failure; no animals were excluded based on phenotype severity or experimental outcome.

### 2.8 Human transcriptomic analysis using NephroSeq

Human glomerular transcriptomic data were retrieved from the NephroSeq platform (v5, University of Michigan). Two independent glomerular microarray datasets were analyzed: Reich IgAN Glom (IgA nephropathy, n = 27; healthy living-donor controls, n = 6) and Ju CKD Glom (focal segmental glomerulosclerosis, n = 25; healthy living-donor controls, n = 21). NephroSeq-normalized expression values for PIK3C3 were used for analysis. Differential expression of PIK3C3 between disease and control groups was assessed using the statistical functions implemented in NephroSeq. Correlation between glomerular PIK3C3 expression and estimated glomerular filtration rate (eGFR) was assessed using Pearson correlation in Reich IgAN Glom samples with available clinical data (n = 26).

## 3 Results

### 3.1 Generation and validation of podocyte-specific *Vps34* knockout mice

**Figure 1:**
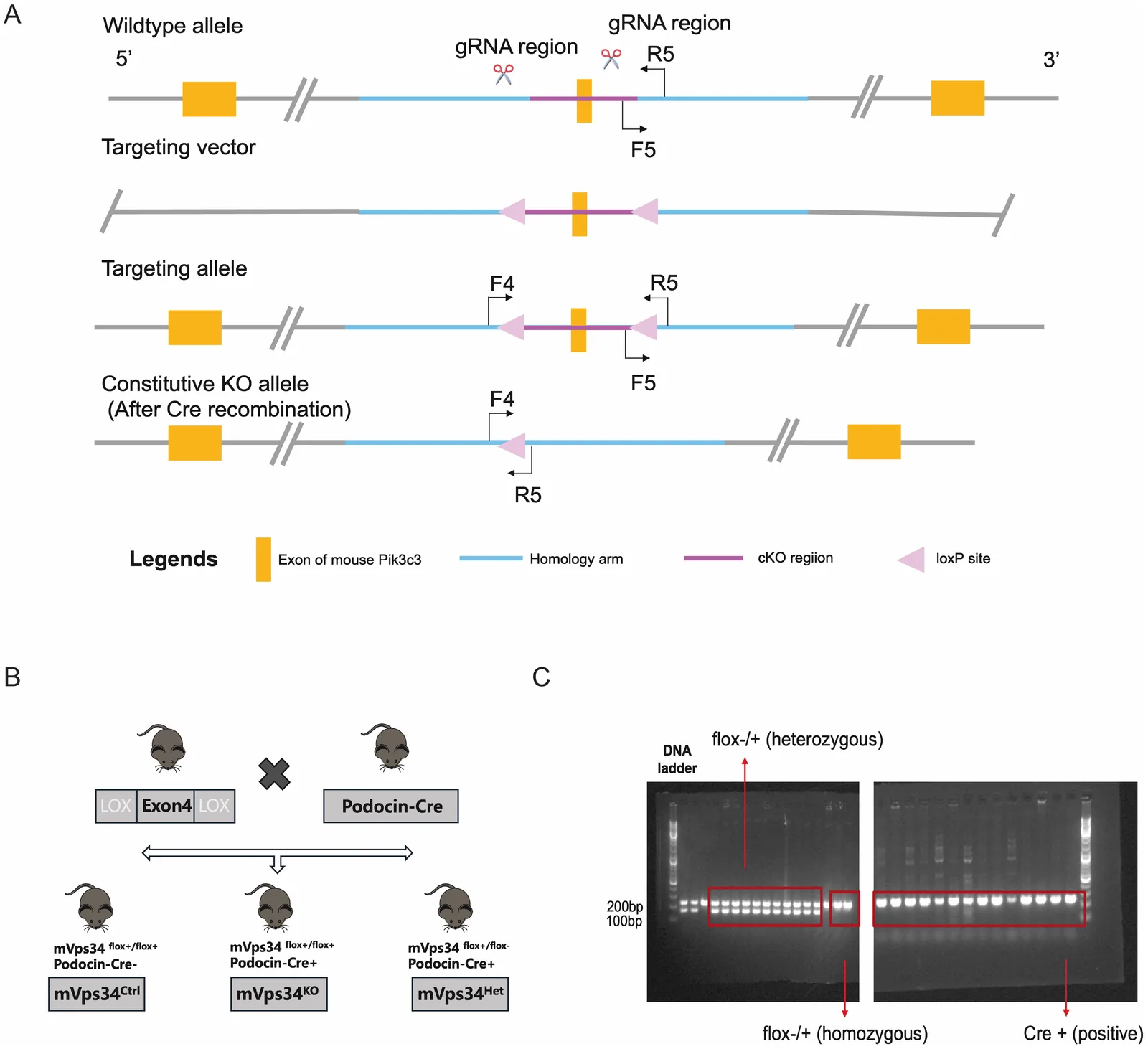
Generation of an mVps34 (*Pik3c3*) conditional knockout mouse and selective deletion in podocytes. **(A)** Schematic representation of the conditional targeting strategy for the *Pik3c3* gene. LoxP sites were inserted flanking exon 4 using CRISPR/Cas9-mediated genome editing. Cre-mediated excision of exon 4 results in a frameshift mutation that abolishes Vps34 kinase activity. **(B)** Podocyte-specific deletion of *Pik3c3* was achieved by crossing *Pik3c3^flox/flox* mice with Podocin*-*Cre transgenic mice. **(C)** Genotyping of offspring confirmed floxed and Cre alleles in podocyte-specific knockout mice (*Pik3c3*^flox/flox; Podocin-Cre^+, designated KO), heterozygous mice (*Pik3c3*^flox/+; Podocin-Cre^+, designated HET), and Cre-negative floxed littermate controls (*Pik3c3*^flox/flox; Podocin-Cre^–, designated FLOX).

To elucidate the function of Vps34 in podocytes, a conditional knockout mouse model was generated by flanking exon 4 of the Pik3c3 gene with loxP sites using CRISPR/Cas9-mediated genome editing. Successful creation of the floxed allele was confirmed by polymerase chain reaction (PCR) and sequencing. Excision of exon 4 induces a frameshift mutation that effectively abolishes Vps34 kinase activity. For podocyte-specific gene ablation, Pik3c3^flox/flox^ mice were crossed with Podocin-Cre (NPHS2-Cre) transgenic mice, in which Cre recombinase is selectively expressed in podocytes beginning at the capillary loop stage of glomerular development. The resulting Pik3c3^flox/flox^; Podocin-Cre^+^ progeny (designated as mVps34^pdKO^) and their Cre-negative littermate controls (Pik3c3^flox/flox^; Podocin-Cre^−^, referred to as mVps34^Ctrl^) were identified by genotyping of tail DNA. Mendelian inheritance patterns were observed at birth (**Figure 1**).

### 3.2 Early-onset glomerular dysfunction and accelerated mortality

**Figure 2:**
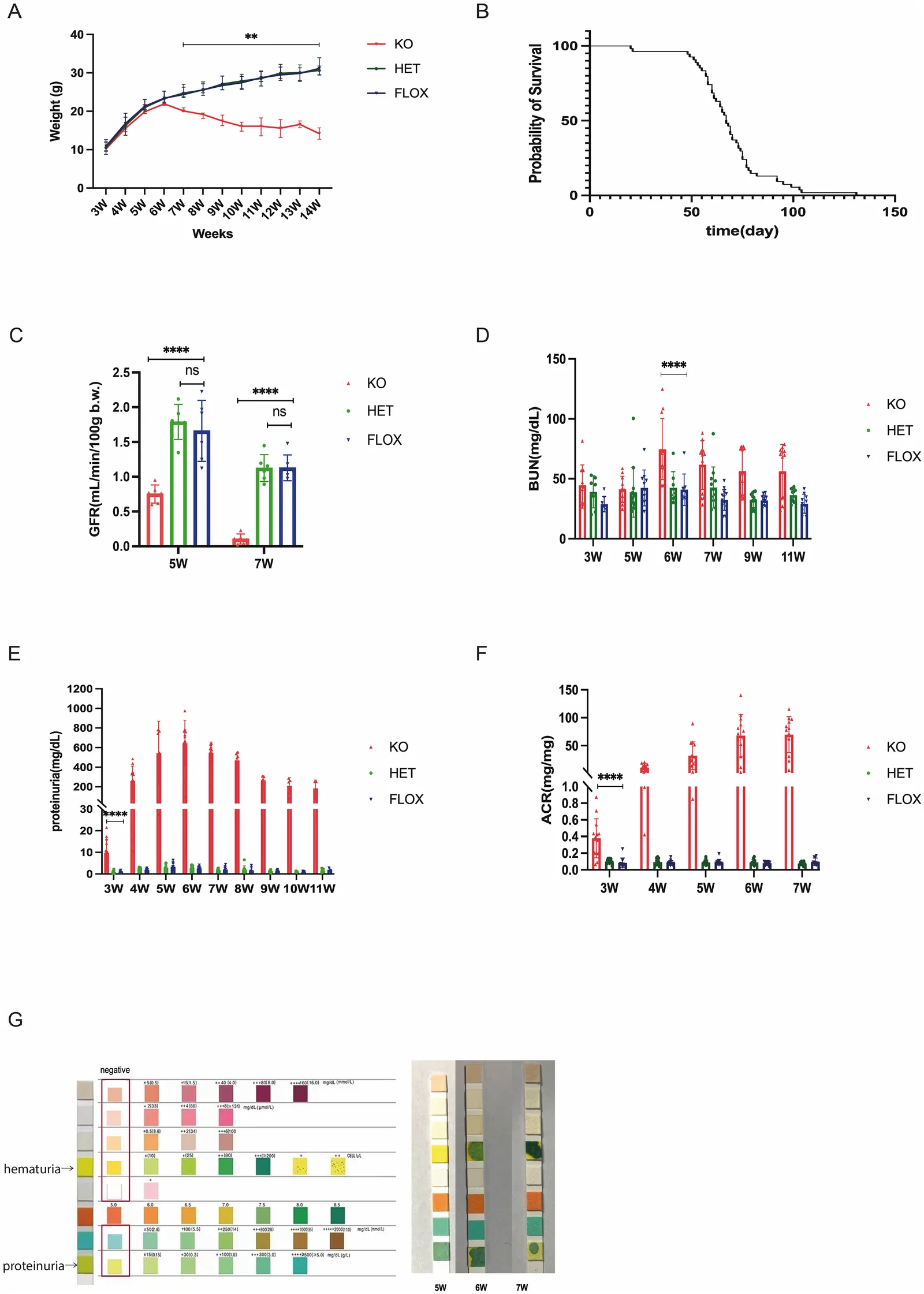
Progressive renal dysfunction and early mortality in podocyte-specific Vps34 knockout mice. **(A)** Body weight of KO (*Pik3c3*^flox/flox; Podocin-Cre^+), HET (*Pik3c3*^flox/+; Podocin-Cre^+), and FLOX (*Pik3c3*^flox/flox; Podocin-Cre^–) mice from 3 to 14 weeks of age (n = 6–8 per group). **(B)** Kaplan–Meier survival curves of KO mice (n = 54). **(C)** Estimated glomerular filtration rate (eGFR) at 5 and 7 weeks of age (n = 6 per group). **(D)** Blood urea nitrogen (BUN) levels at 3–11 weeks of age (n = 5–7 per group). **(E)** Quantification of proteinuria from 3 to 11 weeks of age (n = 6–13 per group). **(F)** Urinary albumin-to-creatinine ratio (ACR) at 3–7 weeks of age (n = 6 per group). **(G)** Representative urine dipstick tests showing proteinuria and hematuria at 5–7 weeks of age. Values are means ± SEM. **P < 0.01; ****P < 0.0001; NS, not significant.

Homozygous mVps34^pdKO^ mice were born at expected Mendelian ratios and exhibited no overt postnatal abnormalities during the initial two weeks of life (n = 54). However, by four weeks of age, growth retardation became evident—mVps34^pdKO^ mice displayed significantly reduced body weight (15.1 ± 1.8 g) relative to littermate controls (17.4 ± 2.0 g, n = 8). By seven weeks, weight reduction approximated 10% as compared to controls (**Figure 2A**).

Survival analysis revealed accelerated mortality in the mVps34^pdKO^ cohort. Kaplan–Meier curves demonstrated that mortality commenced as early as six weeks, with a precipitous survival decline thereafter. The median survival for mVps34^pdKO^ mice was nine weeks; by twelve weeks, survival fell below 20%, and all mutants had succumbed by eighteen weeks. No deaths occurred among control groups over the same period (**Figure 2B**).

Renal functional decline was evident in mVps34^pdKO^ mice. Non-invasive assessment by transcutaneous fluorescence-based monitoring of FITC-sinistrin clearance revealed that estimated glomerular filtration rate (eGFR) was markedly diminished by five weeks in pdKO mice (0.753 ± 0.053 mL·min^-1^·100 g^-1^ body weight, n = 6) compared with floxed littermate controls (1.660 ± 0.179 mL·min^-1^·100 g^-1^ body weight, n = 6; P < 0.0001), indicating early impairment of glomerular filtration capacity (**Figure 2C**). By seven weeks, renal dysfunction had progressed further, with eGFR decreasing to 0.105 ± 0.030 mL·min^-1^·100 g^-1^ body weight in pdKO mice (n = 6), compared with 1.128 ± 0.076 mL·min^-1^·100 g^-1^ body weight in floxed controls (n = 6; P < 0.0001) (**Figure 2C**).

Consistent with this, serum biochemical analysis revealed a marked elevation of BUN beginning at 6 weeks (84.89 ± 25.76 mg/dL in pdKO vs 44.21 ± 15.37 mg/dL in controls, p<0.01, n=6-8), as renal dysfunction became more severe at later stages (**Figure 2D**). These data confirm that the onset of glomerular filtration dysfunction precedes overt azotemia, delineating a clear trajectory toward progressive renal failure.

Urinary abnormalities emerged even earlier than serum biochemical changes in mVps34^pdKO^ mice. Proteinuria was observed as early as 3 weeks of age, immediately following weaning, with mean urinary protein levels of 9.82 ± 6.20 mg/dL (n=13), compared with minimal levels in littermate controls (p<0.01, **Figures 2E, F**). Proteinuria then rose sharply after 4 weeks, reaching nephrotic-range levels by 5–6 weeks, and remained persistently elevated thereafter. In contrast, heterozygous and floxed controls maintained stable low urinary protein levels throughout the observation period.

Hematuria was not observed at this stage but became evident by 6 weeks, coinciding with rising BUN and declining eGFR. By 7 weeks, pdKO mice developed overt hematuria (+++ on dipstick testing) (**Figure 2G**). No hematuria was detected in floxed littermate controls at any examined time point. These findings indicate that proteinuria represents the earliest sign of glomerular barrier disruption in pdKO mice, whereas hematuria appears later alongside renal functional decline.

Together, these findings indicate that podocyte-specific loss of Vps34 leads to early-onset glomerular dysfunction and rapidly progressive glomerular injury, characterized by renal failure and early death. The disease progression timeline reveals an essential role of Vps34 in maintaining glomerular filtration barrier integrity and glomerular function during postnatal development.

### 3.3 Progressive glomerulopathy and immune complex deposition

**Figure 3:**
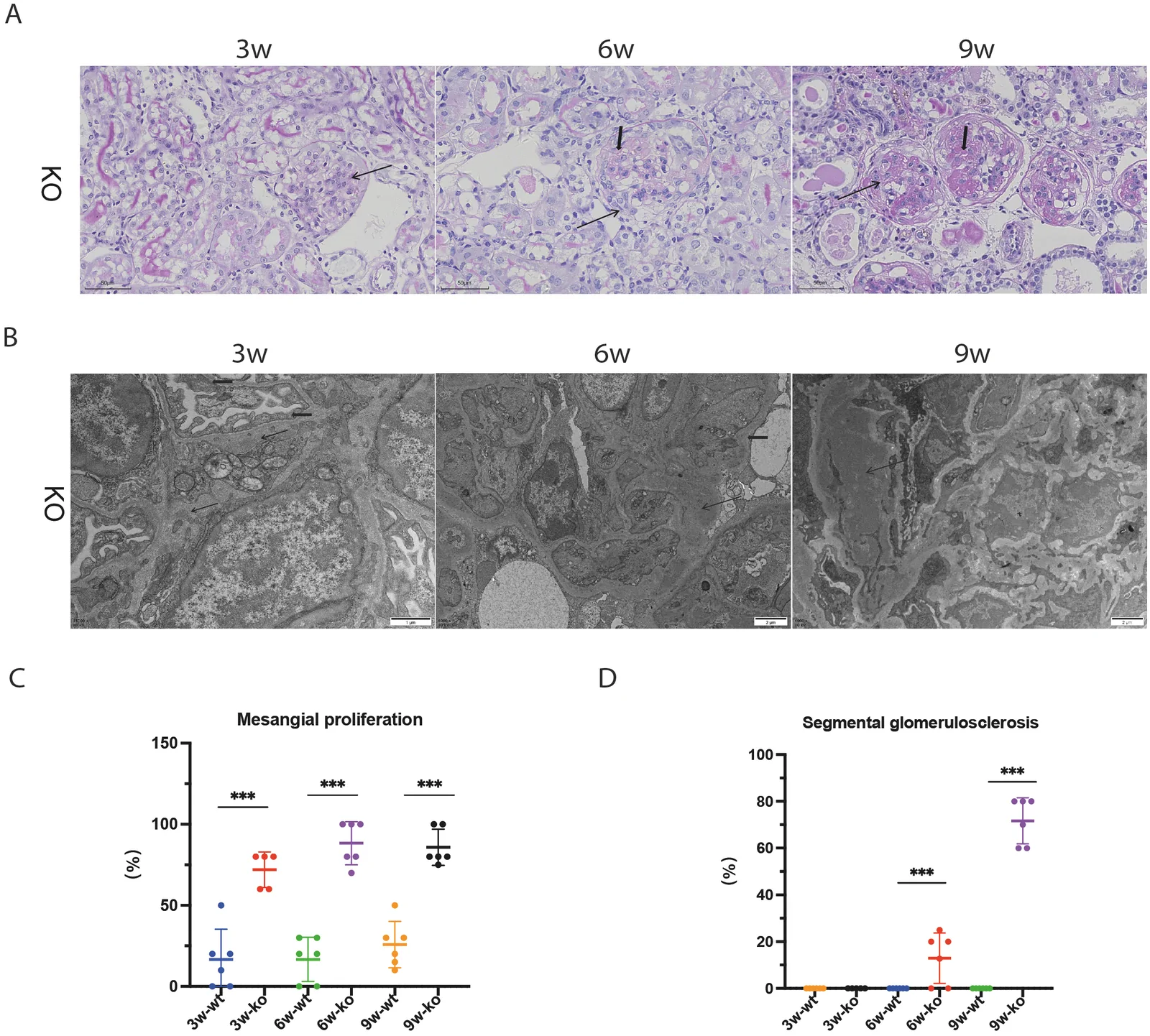
Histopathological and ultrastructural changes in podocyte-specific Vps34 knockout mice. **(A)** Periodic acid–Schiff (PAS) staining of KO (*Pik3c3*^flox/flox; Podocin-Cre^+), kidney sections. At 3 weeks, mesangial expansion was observed (thin arrow). At 6 weeks, glomeruli exhibited segmental sclerosis (thick arrow) and crescent formation (thin arrow). At 9 weeks, global glomerulosclerosis (thin arrow) and segmental sclerosis (thick arrow) were present. Scale bars are indicated in each panel. **(B)** Transmission electron microscopy (TEM) images of KO glomeruli. At 3 weeks, podocytes exhibited foot process effacement (thick arrows) and electron-dense deposits (thin arrows). At 6 weeks, podocytes showed widespread foot process effacement with mesangial electron-dense deposits. At 9 weeks, glomeruli displayed sclerosis with prominent mesangial electron-dense deposits. **(C)** Quantification of mesangial expansion on PAS-stained kidney sections, expressed as the percentage of glomeruli showing mesangial expansion. **(D)** Quantification of segmental glomerulosclerosis, expressed as the percentage of glomeruli exhibiting segmental sclerosis. The per-animal value was used as the statistical unit. Data are presented as mean ± SEM (n = 5 mice for 3 weeks and n = 6 mice for other time points per genotype). Statistical comparisons were performed using the two-tailed Mann–Whitney U test. ***P < 0.001.

Histological analysis revealed a progressive course of glomerular injury in mVps34^pdKO^ mice. At 3 weeks, light microscopy showed early mesangial expansion, evident as widening of the mesangial areas with no overt sclerosis. By 6 weeks, lesions became more complex: focal segmental sclerosis was present in multiple glomeruli, together with persistent mesangial expansion, segmental endothelial swelling, and occasional crescent formation. Mild tubular atrophy with interstitial fibrosis was also observed, involving less than 25% of the renal cortex. At 9 weeks, virtually all glomeruli were affected, with widespread focal segmental sclerosis and some progressing to global glomerulosclerosis. Tubulointerstitial damage was also severe at this stage, with tubular atrophy and interstitial fibrosis extending to more than 50% of the cortex (**Figure 3A**). These findings indicate a temporal progression from early mesangial alterations to extensive glomerulosclerosis and tubulointerstitial remodeling in pdKO mice.

Transmission electron microscopy (TEM) provided ultrastructural evidence of progressive podocyte injury in mVps34^pdKO^ mice. As early as 3 weeks, podocyte foot process effacement and electron-dense deposits were observed (**Figure 3B**). With disease progression, foot process effacement became more extensive and was accompanied by thickening and irregularity of the glomerular basement membrane. In addition, electron-dense deposits were consistently observed within the mesangial and subendothelial regions, features characteristic of immune complex–mediated glomerular injury. Together, these ultrastructural alterations confirm that podocyte-specific Vps34 loss not only compromises podocyte integrity but also promotes the development of immune complex deposition within the glomerular compartment.

**Figure 4:**
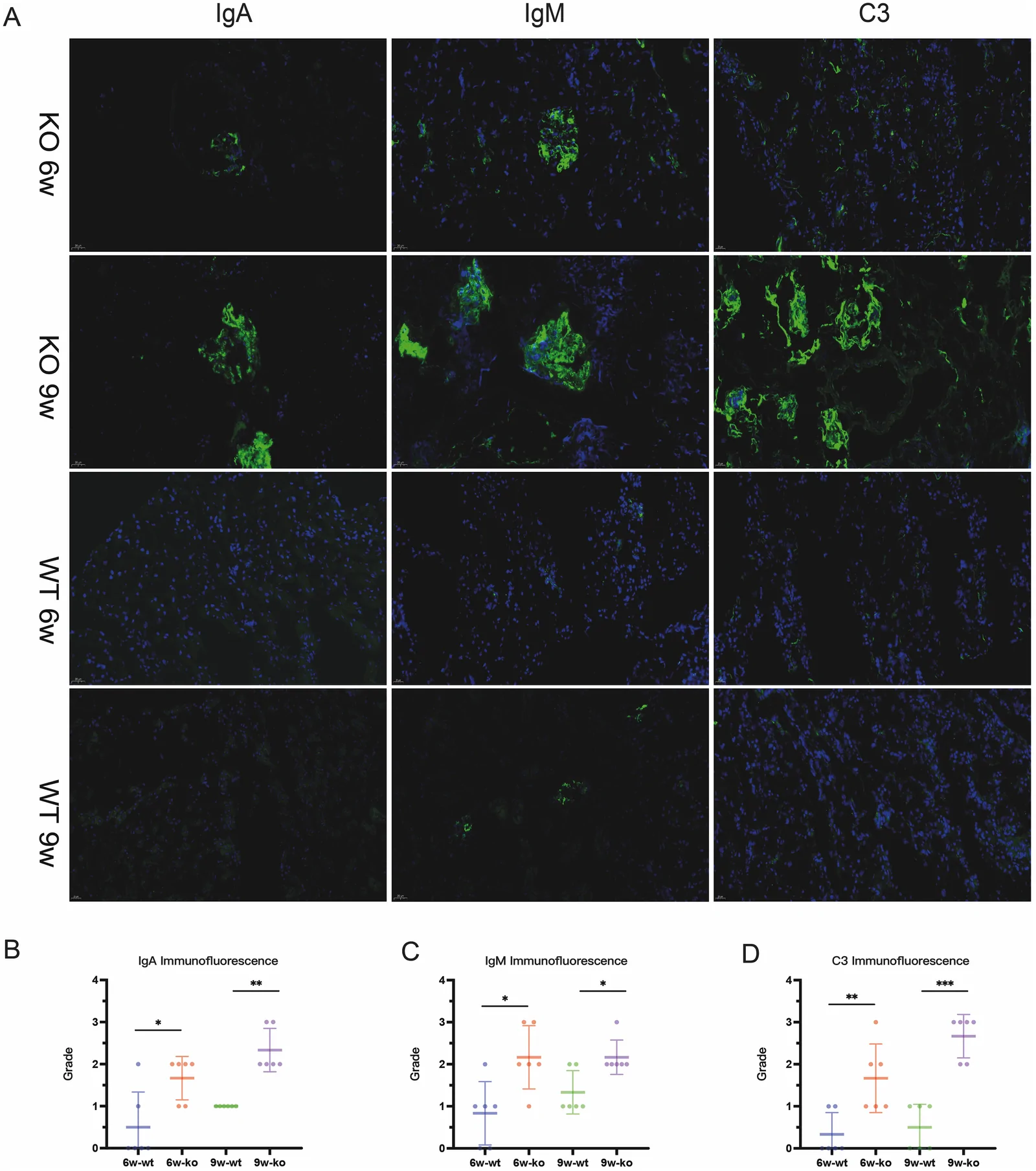
Immunofluorescence staining of glomeruli for IgA, IgM, and C3. **(A)** Immunofluorescence staining of glomeruli for IgA, IgM, and C3. **(B–D)** Semi-quantitative scoring of glomerular IgA **(B)**, IgM **(C)**, and C3 **(D)** deposition based on a 0–4 scale (0 = absent, 1 = trace/segmental faint, 2 = mild diffuse, 3 = moderate diffuse, 4 = strong diffuse). For each mouse, 15 cortical glomeruli were evaluated by an observer blinded to genotype, and the mean score per animal was used for statistical analysis. Data are presented as mean ± SEM (n = 6 mice per genotype per time point). Statistical comparisons were performed using the two-tailed Mann–Whitney U test. *P < 0.05; **P < 0.01; ***P < 0.001.

Immunofluorescence analyses further supported a progressive, IgA-dominant pattern of mesangial immune-complex deposition. At 6 weeks, mesangial deposits of IgA, IgM and complement C3 were readily discernible, and by 9 weeks these deposits were markedly more intense and diffuse, consistent with an established immune-complex glomerulopathy (**Figure 4A**). Semi-quantitative scoring (0–4 scale, 15 glomeruli/mouse, n = 6 mice/group, blinded) confirmed significantly higher intensities of mesangial IgA, IgM and C3 in mVps34^pdKO^ mice than in floxed littermate controls at both 6 and 9 weeks (all P < 0.05; **Figures 4B–D**). Together, these data document a temporally staged progression of mesangial immune-complex deposition — predominantly IgA — that is temporally associated with, but does not by itself prove causality between, podocyte Vps34 loss and mesangial immune-complex accumulation.

### 3.4 Systemic immunoglobulin dysregulation

**Figure 5:**
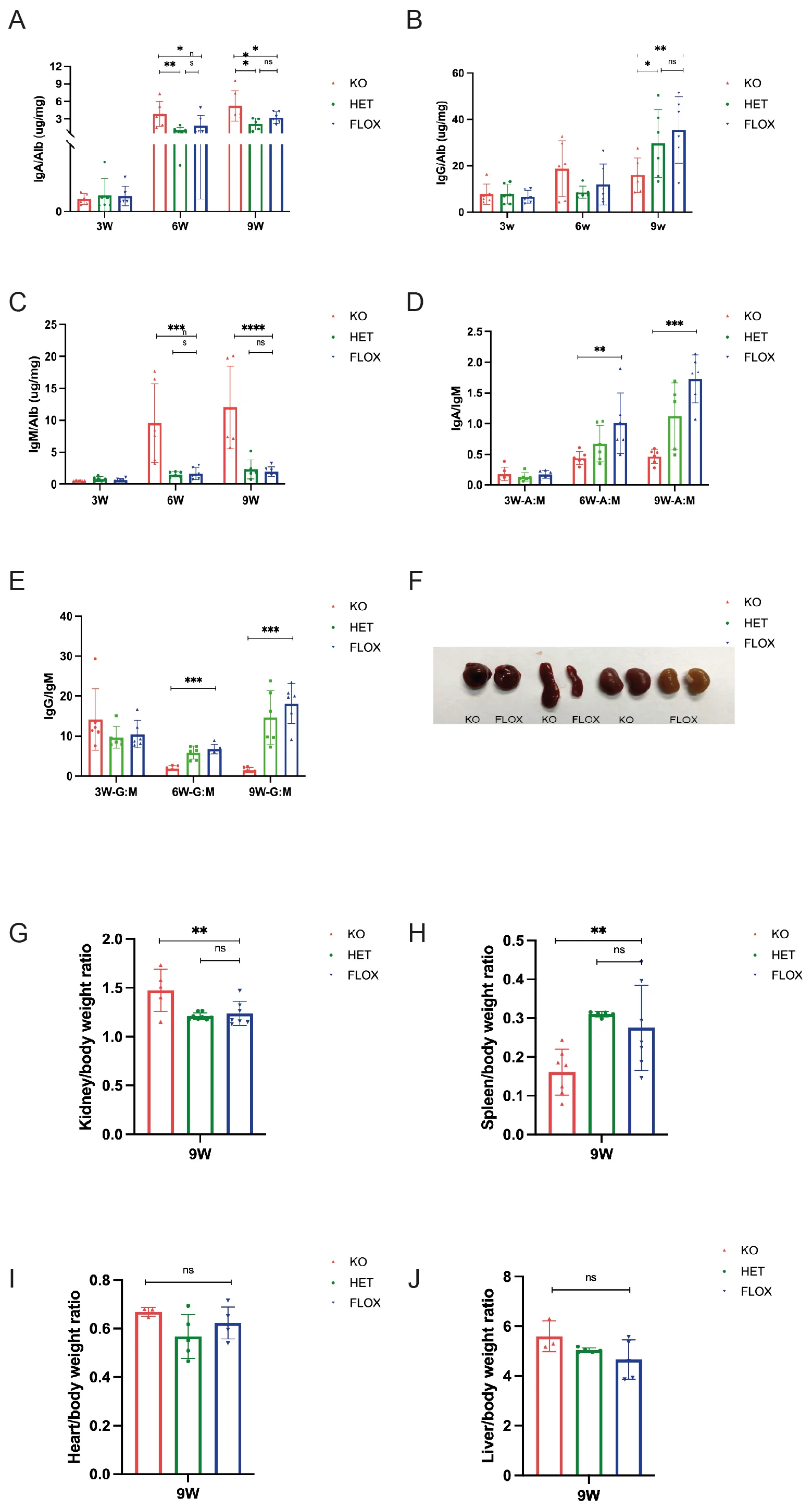
Serum immunoglobulin levels and organ-to-body weight ratios in podocyte-specific Vps34 knockout mice. **(A–E)** Serum immunoglobulin levels in KO (Pik3c3^flox/flox; Podocin-Cre^+), HET (Pik3c3^flox/+; Podocin-Cre^+), and FLOX (Pik3c3^flox/flox; Podocin-Cre^–) mice at 3, 6, and 9 weeks of age. **(A)** Serum IgA levels normalized to albumin (IgA/Alb). **(B)** Serum IgG levels normalized to albumin (IgG/Alb). **(C)** Serum IgM levels normalized to albumin (IgM/Alb). **(D)** IgA/IgM ratio. **(E)** IgG/IgM ratio. **(F–J)** Organ-to-body weight ratios in KO and FLOX mice at 9 weeks of age. **(F)** Representative images of heart, spleen, and kidney **(G)** Spleen-to-body weight ratio. **(H)** Kidney-to-body weight ratio. **(I)** Liver-to-body weight ratio. **(J)** Heart-to-body weight ratio. Values are means ± SEM. *P < 0.05; **P < 0.01; ***P < 0.001; **P < 0.0001; NS, not significant. Sample sizes are indicated by the individual data points in each panel.

To assess whether podocyte-specific Vps34 deficiency influences systemic immunoglobulin production, we quantified serum IgA, IgM, and IgG levels at 3, 6, and 9 weeks and normalized to albumin concentrations to account for hypoalbuminemia. At 3 weeks, immunoglobulin levels were comparable across genotypes. By 6 weeks, however, mVps34pdKO mice exhibited an increase in serum IgA and IgM compared with heterozygous and floxed controls (p<0.01). At 9 weeks, serum IgA and IgM were markedly elevated, whereas IgG was significantly reduced in pdKO mice (all p<0.01), indicating an altered immunoglobulin distribution (**Supplementary Figure 1**). Even after normalization for hypoalbuminemia, KO mice showed disproportionately high IgA/Alb and IgM/Alb ratios, as well as reduced IgG/Alb (**Figures 5A–C**). Immunoglobulin ratio analysis further showed an increase in the IgA/IgM ratio and a decrease in the IgG/IgM ratio in KO mice from 3 to 6 weeks, with these alterations persisting at 9 weeks (**Figures 5D, E****).** Collectively, these findings demonstrate that podocyte-specific Vps34 deficiency leads to systemic alterations in circulating immunoglobulin profiles.

### 3.5 Altered organ-to-body weight ratios in mVps34pdKO mice

Consistent with growth retardation, mVps34^pdKO^ mice exhibited significant alterations in organ mass ratios by 9 weeks of age (n=6–8 per group). The kidney-to-body weight ratio was significantly increased in pdKO mice (1.48 ± 0.22) compared with heterozygous (1.21 ± 0.04, p<0.01) and floxed controls (1.24 ± 0.12, p<0.01), reflecting renal hypertrophy secondary to progressive injury (**Figures 5F, G**). The spleen-to-body weight ratio was markedly reduced in mVps34^pdKO^ mice (0.16 ± 0.06) compared with heterozygous (0.31 ± 0.01, p<0.01) and floxed controls (0.28 ± 0.11, p<0.01) (**Figure 5H**), whereas no significant differences were observed in heart- or liver-to-body weight ratios among genotypes (**Figures 5I, J**).

### 3.6 Transcriptomic trajectory: immune activation and fibrosis

**Figure 6:**
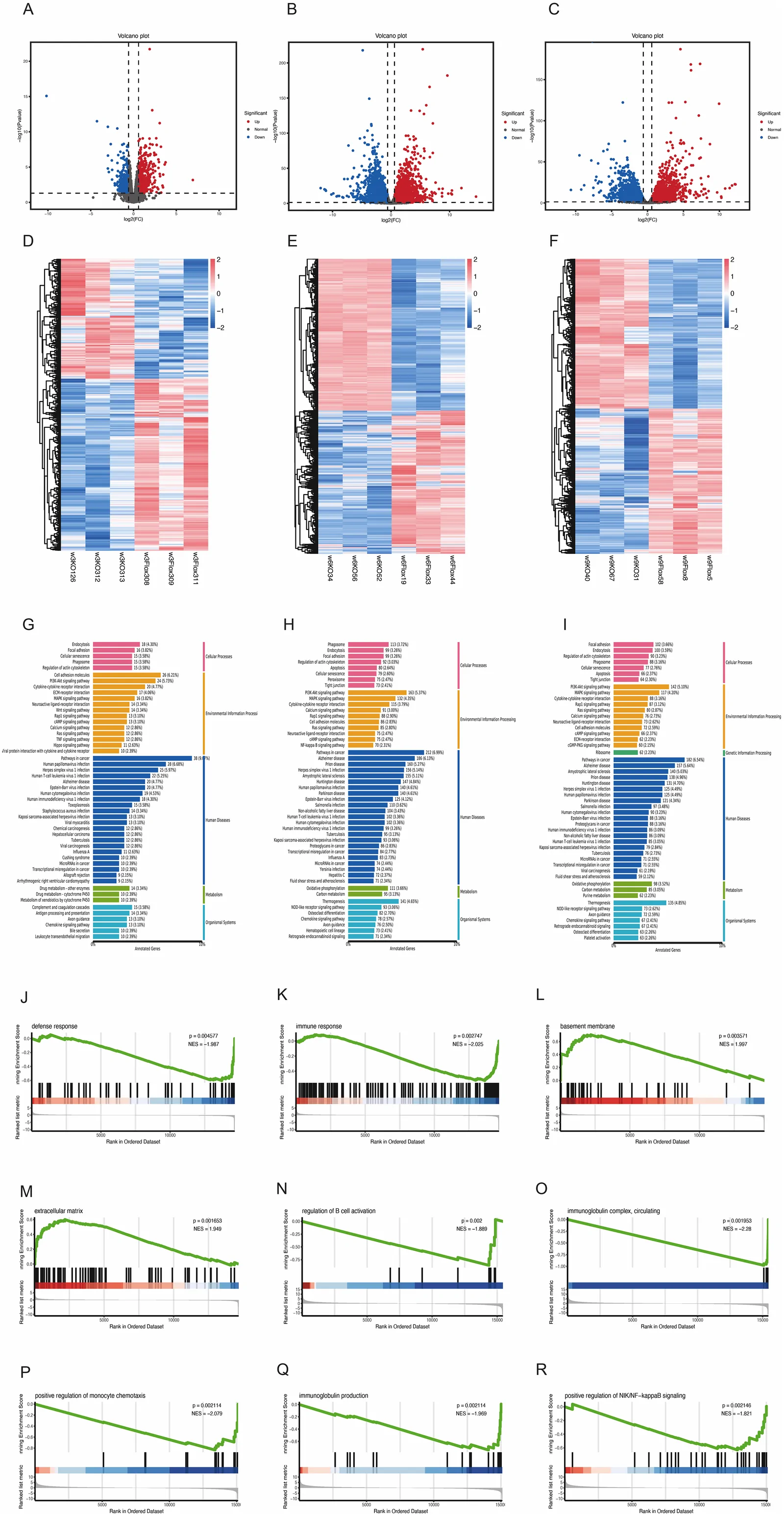
Whole-kidney bulk transcriptomic profiling of podocyte-specific Vps34 knockout mice. **(A–C)** Volcano plots showing differentially expressed genes (DEGs) in KO (*Pik3c3*^flox/flox; Podocin-Cre^+) versus FLOX (*Pik3c3*^flox/flox; Podocin-Cre^–) kidneys at 3 weeks **(A)**, 6 weeks **(B)**, and 9 weeks **(C)**. **(D–F)** Heatmaps of representative DEGs in KO and FLOX kidney at 3 weeks **(D)**, 6 weeks **(E)**, and 9 weeks **(F)**. **(G–I)** KEGG pathway enrichment analysis of DEGs from KO and FLOX kidney at 3 weeks **(G)**, 6 weeks **(H)**, and 9 weeks **(I)**. Gene set enrichment analysis (GSEA) of biological processes and cellular components at 3 weeks **(J–L)**, immune-related pathways at 6 weeks **(M–P)**, and signaling and inflammatory pathways at 9 weeks **(Q–R)**.

Bulk RNA-sequencing of whole-kidney tissue from mVps34pdKO and littermate control mice across time points uncovered stage-specific gene-expression changes (**Figures 6A–F**). At 3 weeks, KEGG pathway analysis revealed enrichment of antigen processing and presentation, leukocyte transendothelial migration, and complement and coagulation cascades (**Figure 6G**). Gene set enrichment analysis (GSEA) further highlighted biological processes related to host defense and immune activation, while cellular component terms were enriched for basement membrane and extracellular matrix organization (**Figures 6J–M**).

By 6 weeks, the number of differentially expressed genes (DEGs) expanded substantially (**Figure 6H**). Pathway analysis consistently identified cytokine–cytokine receptor interactions, B cell activation, and immunoglobulin complex formation as significantly upregulated (**Figures 6N–P**). In parallel, ECM-related pathways—including extracellular matrix organization and focal adhesion—consistently upregulated, indicative of active fibrotic remodeling of the glomerular microenvironment.

At 9 weeks, the transcriptomic profile was dominated by persistent immune activation and amplified inflammatory signaling. KEGG analysis demonstrated sustained enrichment of focal adhesion and cytokine signaling pathways (**Figure 6I**), while GSEA revealed increased expression of gene sets involved in NF-κB activation, monocyte chemotaxis, and immunoglobulin production (**Figures 6Q, R**).

Collectively, these findings delineate a temporal trajectory of transcriptomic dysregulation in Vps34-deficient kidneys, progressing from early immune disturbance at 3 weeks to pronounced immune activation and matrix remodeling by 6–9 weeks. The observed mesangial IgA predominance is temporally associated with podocyte dysfunction, providing insight into the interaction between podocyte injury and immune-complex glomerular injury and establishing a genetic model for studying glomerular immune crosstalk.

### 3.7 Inflammatory and DAMP-related gene signatures in Vps34-deficient kidneys

**Figure 7:**
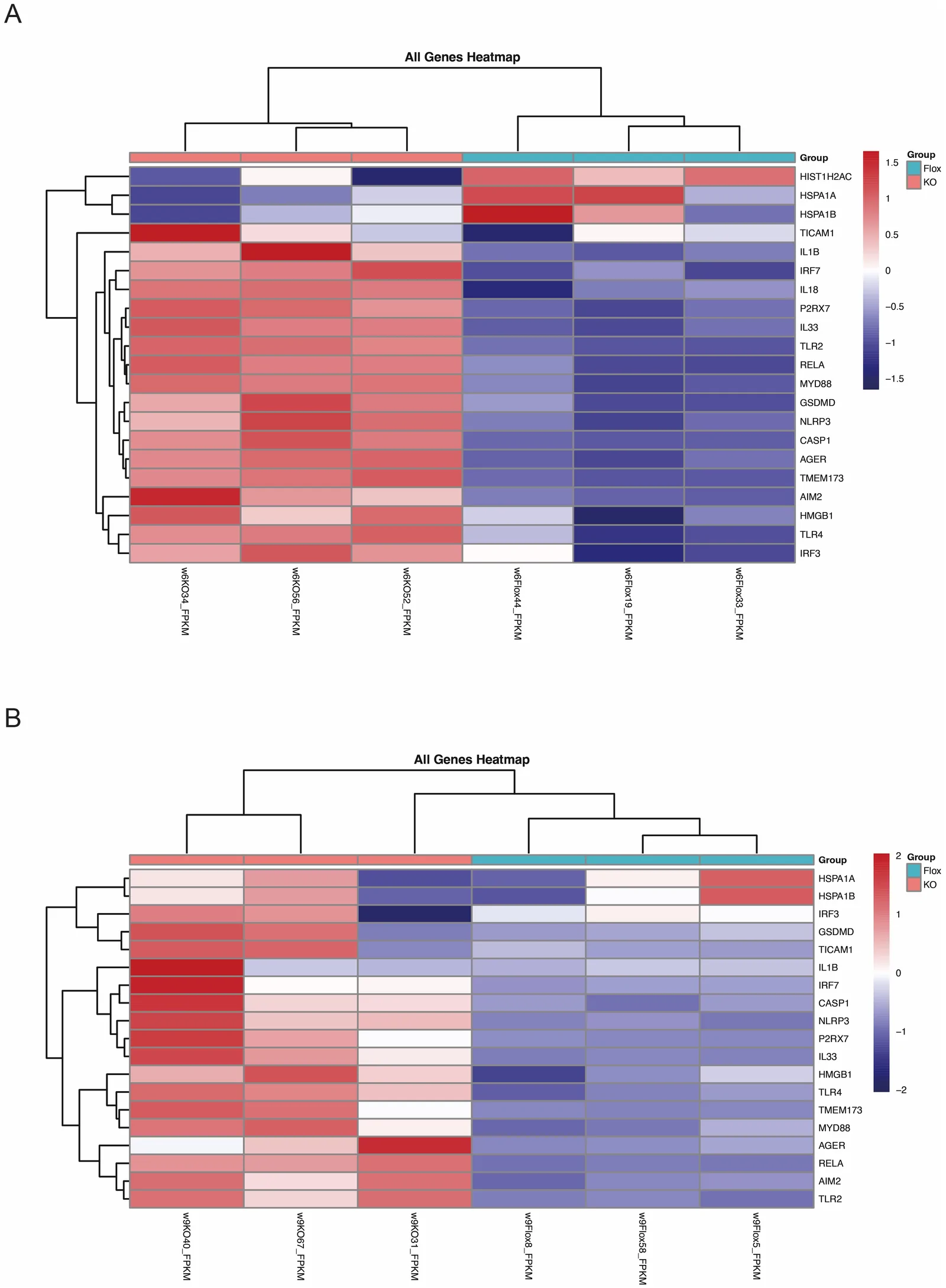
Expression of DAMP- and inflammasome-related genes in whole-kidney transcriptomes from podocyte-specific Vps34 knockout mice. **(A, B)** Heatmap of representative DAMP sensors, pattern recognition receptors and downstream effectors in KO (Pik3c3^flox/flox; Podocin-Cre^+) and FLOX (Pik3c3^flox/flox; Podocin-Cre^–) kidney at 6 weeks **(A)** and 9 weeks **(B)** of age. Gene expression values were log-transformed and z-score normalized across samples.

To further refine the immune-related transcriptomic changes identified above, we next focused on inflammatory and innate immune gene signatures. Differential expression analysis identified significant upregulation of multiple damage-associated molecular pattern (DAMP)– and inflammasome-related genes in mVps34pdKO kidneys (**Figures 7A, B**). Canonical DAMPs, including Hspa1a/b, Il33, and P2rx7, were among the significantly upregulated genes, together with their associated pattern recognition receptors and sensors (Tlr2, Tlr4, Ager, Tmem173, Aim2), as well as downstream inflammasome-related effectors (Myd88, Irf7, Nlrp3, Casp1, Il1b, Gsdmd), all of which showed significant differential expression (adjusted p < 0.05 and |log2FC| > 1).

## 4 Discussion

This study establishes a podocyte-selective Pik3c3 knockout mouse in which loss of Vps34 causes early-onset proteinuria and progressive glomerulosclerosis, consistent with previous Vps34 podocyte-specific models. Beyond this established podocytopathy phenotype, our study identifies an IgA-dominant immune-complex deposition pattern with IgM and C3 co-deposition, systemic alterations in circulating immunoglobulin composition, and disease-stage-dependent changes in immune- and matrix-related transcriptional programs in whole-kidney tissue. These findings suggest a potential link between podocyte Vps34-dependent vesicular homeostasis and alterations of the glomerular immune microenvironment in proteinuric kidney disease.

Three lines of evidence support this interpretation. First, the temporal sequence in mVps34^pdKO mice — proteinuria and podocyte ultrastructural injury by 3 weeks, followed by progressive mesangial IgA, IgM and C3 deposition at 6 and 9 weeks, and subsequent glomerulosclerosis — indicates that podocyte injury precedes detectable immune-complex deposition. Second, the podocyte-restricted genetic deletion (Nphs2-Cre × Pik3c3^flox/flox) indicates that this phenotype develops without a genetically introduced defect in circulating immune cells, mesangial cells, or complement components. Third, whole-kidney transcriptomic analysis revealed progressive changes in immune-, immunoglobulin-, cytokine-, and matrix-related transcriptional programs accompanying disease progression. Together, these observations suggest an association between podocyte Vps34-dependent vesicular homeostasis and alterations in the glomerular immune environment in IgA-dominant immune-complex glomerular injury.

The rapid onset of proteinuria and progressive loss of filtration coincide with severe ultrastructural podocyte injury, sharing canonical features of minimal change disease and focal segmental glomerulosclerosis (18, 19), yet distinguished by the presence of marked mesangial IgA deposition. Together, these findings suggest that podocyte stress and loss of endolysosomal integrity are associated with early local immune activation, indicating that podocyte dysfunction represents an underappreciated component of immune dysregulation in IgA-dominant immune-complex glomerular injury.

Our transcriptomic analyses reveal early upregulation of antigen presentation mechanisms, complement, and B cell activation, a signaling landscape that recapitulates both innate and adaptive immune responses documented in proteinuric kidney disease (20–22). These findings further extend the role of podocyte Vps34 deficiency beyond structural injury by demonstrating an association with immune-related pathway enrichment within the renal microenvironment. Podocytes have been shown to exhibit immune-like properties, including the capacity to interact with immune cells and to modulate the local renal microenvironment through cytokine secretion and expression of pattern recognition receptors (23–25).

Our transcriptomic data also revealed increased expression of DAMP-associated and inflammasome-related genes in Vps34-deficient kidneys, suggesting activation of inflammatory programs (26). However, these transcriptional changes alone do not establish functional DAMP release or inflammasome activation, and the specific cellular sources and functional consequences of these pathways require further investigation.

Beyond inflammatory signaling, podocyte injury may also be accompanied by changes in the expression or distribution of structural proteins with potential immunological relevance. In this study, we observed increased expression of Sptbn1 (encoding βII-spectrin) in Vps34-deficient kidneys (**Supplementary Figure 2**), suggesting early cytoskeletal stress or remodeling during podocyte injury. Notably, βII-spectrin has been reported to become aberrantly exposed on mesangial cell surfaces in gddY mice, a spontaneous model of IgA nephropathy, where it can serve as a target for pathogenic IgA autoantibodies (27). Although the functional consequences of Sptbn1 upregulation were not directly examined in our model, these observations raise the possibility that podocyte structural alterations may contribute to immune recognition in IgA-dominant glomerulopathies (28, 29).

To place these findings in a human context, we interrogated two independent glomerular microarray datasets from the NephroSeq platform. Glomerular PIK3C3 transcript levels tended to be lower in IgA nephropathy than in healthy living donors and were negatively correlated with estimated GFR in the same cohort (**Supplementary Figure 3A**, **3B**), whereas PIK3C3 levels appeared higher in FSGS glomeruli than in controls (**Supplementary Figure 3C**). We interpret this apparently opposite directionality with caution. Both datasets are derived from bulk glomerular microarrays and therefore reflect the mixed cellular composition of the sampled glomeruli, including podocytes, mesangial cells, endothelial cells, and infiltrating immune cells. Thus, disease-associated differences in cellular composition and glomerular remodeling, such as altered podocyte abundance in FSGS, may influence bulk PIK3C3 expression signals. The NephroSeq analysis should therefore be regarded as an exploratory, hypothesis-generating observation indicating that PIK3C3 expression varies across human glomerular diseases in a disease- and cell-context-dependent manner. These findings are consistent with, but not sufficient to establish, a functional role for Vps34 in human glomerular disease. Cell-resolved approaches, including single-nucleus RNA sequencing, spatial transcriptomics, and cell marker-based immunohistochemical analyses, will be required to define the podocyte-specific contribution of these signals.

The translational relevance of these findings lies in highlighting the potential interplay between podocyte biology and immune-complex glomerular disease. In human IgA nephropathy, podocyte injury has often been interpreted as a consequence of mesangial IgA deposition. Our model demonstrates that podocyte-specific disruption of vesicular trafficking is associated with the development of an IgA-dominant mesangial immune-complex phenotype, raising the possibility that primary podocyte dysfunction may contribute to immune-complex glomerular injury in a subset of settings. This concept is consistent with the observation that glomerular PIK3C3 transcript levels vary across human IgA nephropathy and FSGS in a disease- and cell-context-dependent manner (**Supplementary Figure 3**), and with reports that podocyte structural proteins such as βII-spectrin may serve as neo-epitopes associated with pathogenic IgA responses (27).

**Figure 8:**
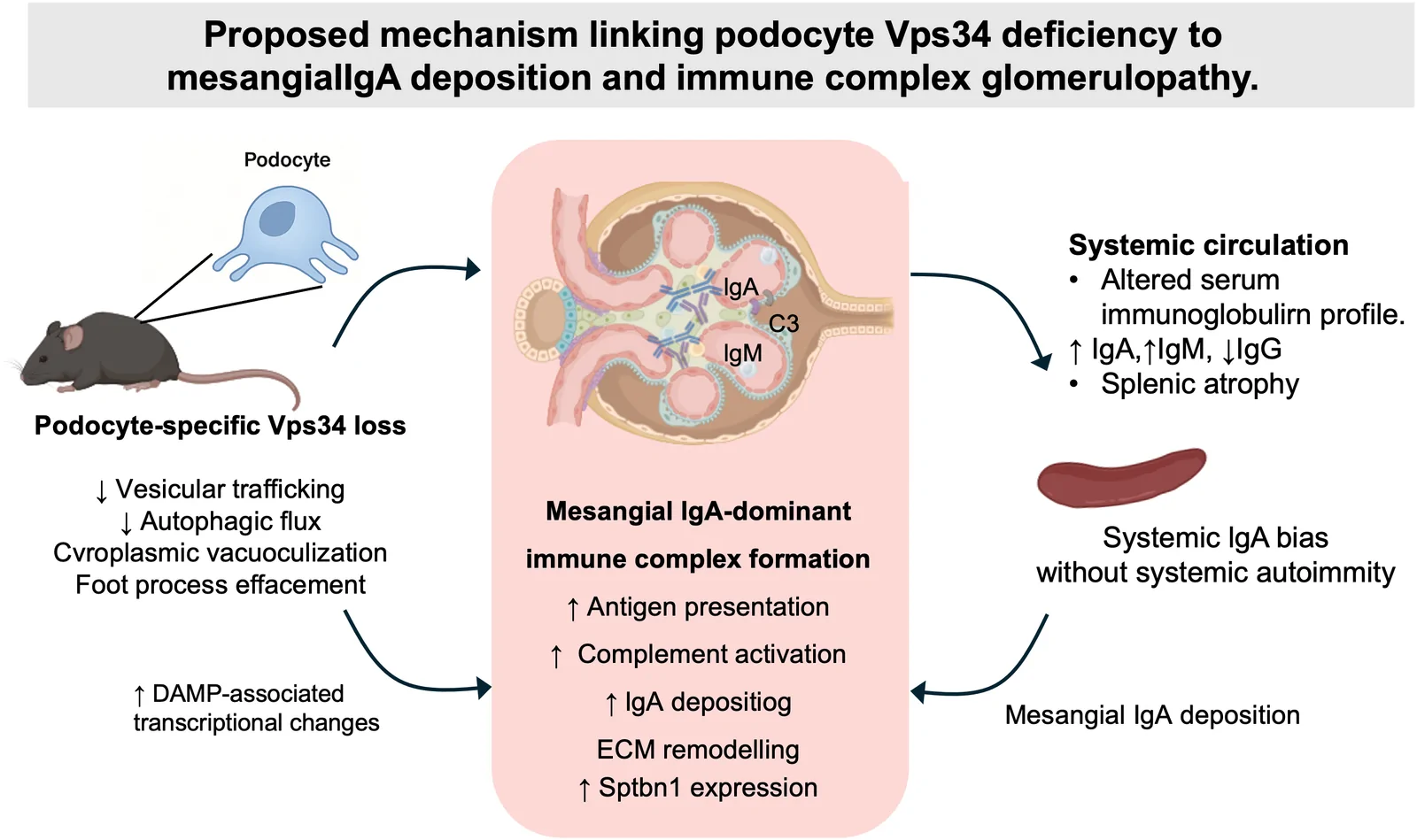
Proposed mechanism linking podocyte Vps34 deficiency to mesangial IgA deposition. Schematic representation of the proposed relationship between podocyte Vps34 deficiency, glomerular immune dysregulation, and mesangial IgA-dominant immune complex deposition.

Based on these observations, we propose a conceptual model in which podocyte-specific loss of Vps34 is associated with immune-related transcriptional changes and mesangial IgA accumulation (**Figure 8**). Although mesangial IgA represents the dominant immunoglobulin deposit in mVps34^pdKO kidneys, we do not claim that this model reproduces the full pathogenesis of human IgA nephropathy. We did not assess galactose-deficient IgA1 or anti–Gd-IgA1 autoantibodies, characterize circulating IgA-containing immune complexes for antigen specificity or pathogenicity, or investigate mucosal immune mechanisms. IgA deposition in this model may partly reflect secondary consequences of severe podocyte injury, altered immunoglobulin handling across a compromised filtration barrier, and generalized inflammatory responses. We therefore describe this model as an IgA-dominant immune-complex glomerulopathy associated with podocyte-intrinsic Vps34 loss — a genetic platform for investigating the relationship between podocyte vesicular-trafficking defects and mesangial immune-complex accumulation — rather than as a complete model of IgA nephropathy.

Several limitations should be noted. First, podocyte-restricted protein-level confirmation of Vps34 depletion (e.g., co-immunostaining of Vps34 with podocyte markers or Western blot analysis of isolated glomeruli) was not performed in this study. The interpretation of podocyte-specific Vps34 loss is based on the established Nphs2-Cre × Pik3c3^flox genetic model (7, 10, 11), together with the reproducible stage-specific podocyte injury phenotype observed here. Reduced Pik3c3 transcript abundance was also detected in whole-kidney RNA-seq. A Nphs2-Cre^+; Pik3c3^+/+ (Cre-only) control cohort was not included and should be addressed in future studies.

Second, transcriptomic analyses were performed on whole-kidney tissue rather than isolated glomeruli or sorted podocytes. Therefore, immune-, inflammatory-, and matrix-related transcriptional signatures likely reflect contributions from multiple renal cell populations, including glomerular resident cells, tubulointerstitial cells, and infiltrating immune cells. Cell-resolved approaches, including glomerular isolation, single-cell/single-nucleus RNA sequencing, and spatial transcriptomics, will be required to define the podocyte-intrinsic component of these signals.

Third, DAMP- and inflammasome-related findings represent transcript-level observations. Protein-level validation (cleaved caspase-1, mature IL-1β, cleaved gasdermin-D, HMGB1/ATP release) and pathway-intervention studies will be required to determine whether inflammasome activation has a functional role in this model.

Fourth, urinary immunoglobulin loss was not directly quantified. Therefore, the observed systemic immunoglobulin alterations cannot independently establish changes in immunoglobulin production or systemic B-cell activation.

Fifth, galactose-deficient IgA1, anti–Gd-IgA1 autoantibodies, circulating IgA-containing immune complexes, and mucosal immune mechanisms were not assessed. The model is therefore described as an IgA-dominant immune-complex glomerulopathy associated with podocyte Vps34 loss rather than a complete model of human IgA nephropathy.

Sixth, the RNA-sequencing cohort (n = 3 per genotype per time point) was modest; therefore, transcriptomic analyses are interpreted as hypothesis-generating. Limitations.

## 5 Conclusions

In a podocyte-selective Pik3c3 knockout mouse, loss of Vps34 was associated with early-onset proteinuria, characteristic podocyte ultrastructural injury, and the development of a progressive IgA-dominant mesangial immune-complex glomerulopathy with IgM and C3 co-deposition. These changes were accompanied by broad, disease-stage-dependent alterations in circulating immunoglobulin composition and by whole-kidney changes in immune-, inflammasome-related, and matrix-associated transcriptional programs. These findings suggest a potential link between defective podocyte vesicular trafficking and alterations in the glomerular immune microenvironment in proteinuric disease, and provide a genetic platform for investigating podocyte–mesangial–immune interactions. They do not, and are not intended to, recapitulate the full pathogenesis of human IgA nephropathy; rather, they extend the recognized biology of podocyte Vps34 beyond cell-intrinsic homeostasis toward a potential role in shaping immune-associated changes within the glomerulus.

## Data Availability

This study does not include large-scale datasets or custom computational algorithms. All data supporting the findings of this work are contained within the article and its **Supplementary Materials**. Additional information regarding the animal models, experimental procedures, or analytical methods used in this study will be made available upon reasonable request. For inquiries related to specific aspects of the research, please contact the corresponding authors X-JZ.
