# Supplementary material for: Podocyte Vps34 deficiency drives early-onset glomerulopathy and mesangial IgA-dominant immune-complex deposition: a novel mouse model linking vesicular trafficking to glomerular immune dysregulation
Source: Front Immunol. 2026 Sep 9;17:1936691. doi: 10.3389/fimmu.2026.1936691 (PMC13597270; doi:10.3389/fimmu.2026.1936691)
Supplement: Supplementary file 1 [file DataSheet1.docx]

**A**

**B**

**C**

**D**

**Supplementary Figure S1. Absolute serum concentrations of albumin, immunoglobulins, and total protein in podocyte-specific Vps34-deficient mice.**

(A–D) Absolute serum concentrations of albumin (Alb, mg/mL), IgA (μg/mL), IgG (mg/mL), and IgM (mg/mL) were measured in podocyte-specific Vps34 knockout mice (KO), heterozygous control mice (HET), and floxed control mice (Flox) at indicated ages (3, 6, and 9 weeks). Absolute immunoglobulin levels are presented without normalization to serum albumin to avoid potential confounding effects caused by severe proteinuria and hypoalbuminemia in KO mice. The data demonstrate progressive alterations in systemic immunoglobulin profiles during disease progression, including increased serum IgM levels in KO mice at later stages. Data are presented as mean ± SD. Each dot represents an individual mouse. Statistical comparisons were performed using one-way ANOVA followed by Tukey’s multiple comparisons test. *P < 0.05, **P < 0.01, ***P < 0.001, ****P < 0.0001; ns, not significant.

**Supplementary Figure 2** (A) Violin plot showing the expression levels of Sptbn1 in KO (Pik3c3^flox/flox; Podocin-Cre^+) and FLOX (Pik3c3^flox/flox; Podocin-Cre^–) glomeruli. Values are means ± SD. *P < 0.05.

**Supplementary Figure 3.** **NephroSeq analysis of PIK3C3 expression in human glomerular diseases**. (A) Glomerular PIK3C3 expression in IgA nephropathy and healthy living donors from the Reich IgAN Glom dataset (IgA nephropathy, n = 27; healthy living donors, n = 6). (B) Correlation between glomerular PIK3C3 expression and estimated glomerular filtration rate (eGFR) in IgA nephropathy samples from the Reich IgAN Glom dataset (n = 26). (C) Glomerular PIK3C3 expression in focal segmental glomerulosclerosis (FSGS) and healthy living donors from the Ju CKD Glom dataset (FSGS, n = 25; healthy living donors, n = 21). Expression values were obtained from NephroSeq-normalized microarray data, and statistical analyses were performed using methods implemented in NephroSeq.

**Supplementary Table 1. Primers used for genotyping of *Pik3c3* conditional knockout mice.**

| **Gene / Target** | **Primer name** | **Sequence (5’–3’)** |
| --- | --- | --- |
| ***Nphs2*** | **NPHS2-F** | **CGGTTATTCAACTTGCACCA** |
| ***Nphs2*** | **NPHS2-R** | **GCGCTGCTGCTCCAG** |
| ***Pik3c3* floxed allele** | **Flox-F5** | **AGACCAACCACTTAAATAGCCCAT** |
| ***Pik3c3* floxed allele** | **Flox-R5** | **GCAGGCTTTTGGGTGTTACTTATT** |
